# A Customized Deep Sleep Recommender System Using Hybrid Deep Learning

**DOI:** 10.3390/s23156670

**Published:** 2023-07-25

**Authors:** Ji-Hyeok Park, Jae-Dong Lee

**Affiliations:** Department of Computer Science, Dankook University, 152 Jukjeon-ro Campus, Suji-gu, Yongin-si 16890, Republic of Korea; pjh72230235@dankook.ac.kr

**Keywords:** recommender system, deep learning, sleep technology, personalized system

## Abstract

This paper proposes a recommendation system based on a hybrid learning approach for a personal deep sleep service, called the Customized Deep Sleep Recommender System (CDSRS). Sleep is one of the most important factors for human life in modern society. Optimal sleep contributes to increasing work efficiency and controlling overall well-being. Therefore, a sleep recommendation service is considered a necessary service for modern individuals. Accurate sleep analysis and data are required to provide such a personalized sleep service. However, given the variations in sleep patterns between individuals, there is currently no international standard for sleep. Additionally, service platforms face a cold start problem when dealing with new users. To address these challenges, this study utilizes K-means clustering analysis to define sleep patterns and employs a hybrid learning algorithm to evaluate recommendations by combining user-based and collaborative filtering methods. It also incorporates feedback top-N classification processing for user profile learning and recommendations. The behavior of the study model is as follows. Using personal information received through mobile devices and data, such as snoring, sleep time, movement, and noise collected through AI motion beds, we recommend sleep and receive user evaluations of recommended sleep. This assessment reconstructs the profile and, finally, makes recommendations using top-N classification. The experimental results were evaluated using two absolute error measurement methods: mean squared error (MSE) and mean absolute percentage error (MAPE). The research results regarding the hybrid learning methods show 13.2% fewer errors than collaborative filtering (CF) and 10.2% fewer errors than content-based filtering (CBF) on an MSE basis. According to the MAPE, the methods are 14.7% more accurate than the CF model and 9.2% better than the CBF model. These results demonstrate that CDSRS systems can provide more accurate recommendations and customized sleep services to users than CF, CBF, and combination models. As a result, CDSRS, a hybrid learning method, can better reflect a user’s evaluation than traditional methods and can increase the accuracy of recommendations as the number of users increases.

## 1. Introduction

Sleep is considered to be one of the most significant factors in modern society that affects human life [1]. As we can see from events like “World Sleep Day,” the importance of sleep is emphasized in our daily lives, and efforts are being made worldwide to prevent and manage sleep disorders. Sleep technology, or “sleep tech,” is recognized as a future industry and is gaining traction, as demonstrated by its inclusion in CES 2020~2022, and it is evolving into various fields through the integration of artificial intelligence and IoT technologies [2,3,4].

Sleep technology, also known as “sleep tech”, is a combination of sleep and technology that aims to improve the quality of sleep by analyzing sleep-related data. The initial steps in this field involved the integration and development of IoT technology [5]. One of the representative products is the motion bed, which collects sleep data from the user and analyzes them to improve the user’s sleep environment [6,7]. Other research directions in sleep technology involve data analysis such as brainwave analysis. Standardized evaluation guidelines are being designed using biological data collected from existing IoT devices, and service satisfaction is being improved through user satisfaction surveys [8]. However, because sleep evaluation guidelines vary between studies and there is no international standard for optimal sleep, it cannot be deemed sufficient to provide accurate sleep state predictions and personalized services. In order to provide personalized sleep services, a requirement for personalized sleep pattern analysis is present [9].

The goal of this research is to analyze non-standardized sleep data in a personalized way using cluster analysis and to solve the cold start problem of the service using a hybrid collaborative filtering approach with profile composition and feedback evaluation. In this paper, we propose a Customized Deep Recommender System (CDSRS) that utilizes motion beds to collect data and construct user profiles and incorporates user feedback evaluations to address the cold start problem. The expected contributions are (i) the improvement of the cold start problem of the platform, (ii) the improvement of the accuracy of hybrid recommendation systems in personalized sleep services, and (iii) the evaluation of pattern recognition through cluster analysis and suitable recommendations for users. These results effectively provide recommendation services for sleep with low relevance and solve the cold start problem that occurs in the platform.

The focus of this study is on the accuracy of the recommendation system. The user profiles, reconstructed using hybrid learning combining pattern definitions and feedback methods, contribute to improving the effectiveness and accuracy of sleep recommendations. Traditional methods, such as CF or CBF, have a problem in that there is no way to increase the accuracy of recommendations as the number of users increases, like those that do not reflect a user’s sleep assessment. However, the CDSRS system is a hybrid learning method that easily reflects a user’s sleep assessment and can increase the accuracy of recommendations as the number of users increases. The evaluation of recommendation accuracy is based on two performance error functions: MSE and MAPE. Section 2 describes related research and technologies, while Section 3 explains the learning method, system structure, and algorithm of CDSRS. Finally, Section 4 and Section 5 describe the research results and conclusions.

## 2. Related Work

### 2.1. Recommender Systems

Recommender systems (RSs) provide necessary and accurate information to help users choose the most suitable option in a particular environment. This has made RSs popular software [10]. They are successfully used by platform-oriented companies such as Netflix and YouTube to provide recommendations to users [11,12]. The goal of an RS is to provide personalized content to users, which can reduce their selection time and increase efficiency. Additionally, when used by many users, RSs can help identify situational needs. There are various types of recommendation algorithms, such as collaborative filtering, content-based filtering, and user-based filtering. However, these types of filtering have a major drawback [13]. When a new user starts using the system, the cold start problem occurs. Many studies have been conducted to address the cold start problem [14,15].

The fields that most use recommendation systems include clothing, movies, and food, among others, and recommendations are provided based on the user’s psychology, personality, and situational information. To provide recommendations, it is important to understand the correlations between this information or apply it according to the RS algorithm. RSs can also provide personalized medicine and digital healthcare in the sleep tech field, helping users improve their health, and can increase the level of predicting and evaluating content by utilizing sleep data [16,17].

### 2.2. Deep Learning (DL)

Deep learning is one of the methods for implementing artificial intelligence, analyzing data, and deriving results based on algorithms. This is why deep learning is popular in building personalized systems [18,19,20]. Deep learning is used in various fields, and machine learning is divided into three types of learning methods: supervised learning, unsupervised learning, and reinforcement learning [21]. Among them, deep learning is a machine learning method that uses neural networks. Current research shows that machine learning techniques and filtering techniques are used in recommendation systems. Hybrid methods that can use deep learning to improve recommendations are also available [22,23].

### 2.3. Sleep Technology

Sleep is an essential element of human life, but it can be overlooked in modern times, leading to sleep disorders. For this reason, sleep tech is gaining popularity, and a significant amount of research is being conducted [24,25,26]. Recent techniques in sleep include sleep posture recognition using microwave Doppler radar and machine learning classifiers and sleep posture recognition using dual-frequency microwave Doppler radar [27,28,29,30,31,32]. However, since there is no standard for sleep data, it is difficult for recommendation systems to provide accurate content. As sleep patterns define machine learning and recommendation system performance, further research is needed. In this paper, the main goal is to create a standard using pattern analysis based on cluster characteristics to improve system efficiency using machine learning.

### 2.4. Personalized Systems

The fourth industrial revolution has led to innovative technological advancements such as cloud computing and artificial intelligence, but it has also given rise to the important element of personalized systems [33]. One of the most prominent examples is personalized recommendation systems. These systems provide tailored recommendation services based on a user’s information. When users are satisfied, the quality of the service improves, leading to expansion into various fields.

## 3. The Proposed CDSRS (Customized Deep Sleep Recommender System)

**Figure 1:**
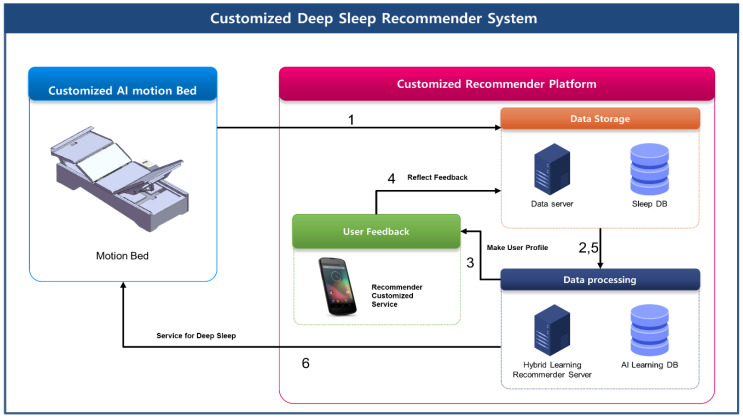
Customized Deep Sleep Recommender System (CDSRS).

The Customized Deep Sleep Recommender System (CDSRS) process is carried out according to steps 1 to 6 shown in Figure 1. In step 1, it collects sleep information using a customized AI motion bed and transmits the data necessary to create a personal profile. In step 2, the data server in the data store is used to configure the user’s profile and sleep content, and step 3 reconstructs the user’s profile when evaluation information is sent. The hybrid learning method in data processing learns algorithms based on user profiles and user evaluations. In step 4, user feedback provides user profiles and collects user evaluation information, which is then transmitted to the data server. The feedback function helps improve the accuracy of sleep recommendations for users. In step 6, it provides sleep services using motion beds to users through recommendations.

**Figure 2:**
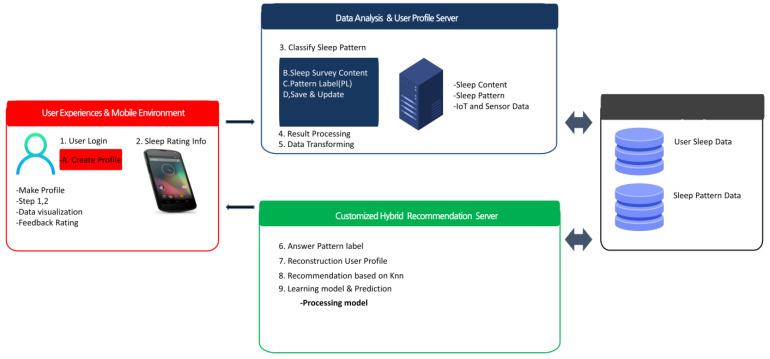
CDSRS service model and service flow steps.

**Figure 3:**
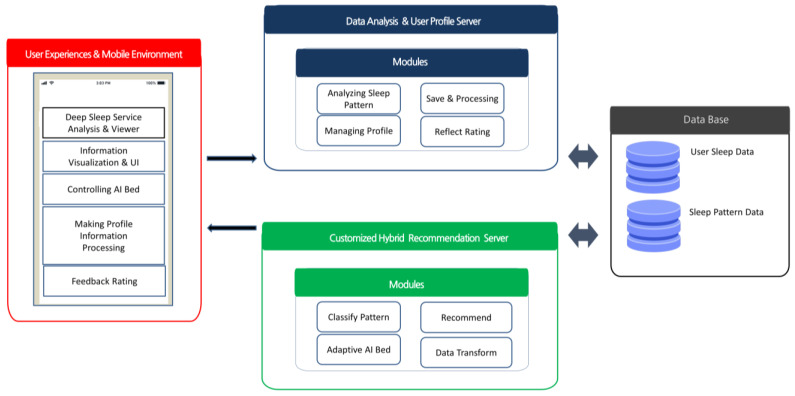
System architecture of the CDSRS.

The sleep system is composed of user sleep data and recommendation information, which are important parts of sleep care. Figure 2 and Figure 3 illustrate the concept of technological development for sleep care design models, system overview, structure, and service utilization cases in the service flow. Figure 2 shows the concept of the service model and introduces the components of each service.

The model consists of nine steps in sequential order. Clustering is performed in steps 3 to 5 using the user’s sleep data to define patterns. Currently, the user’s sleep data are rearranged into patterns. Steps 6 to 9 express detailed sleep patterns by adding user satisfaction data through additional surveys. The user’s satisfaction data, obtained through the survey, are used to respecify the recommendation presented to the user. The CDSRS uses a hybrid learning method to learn and test the dataset, evaluate the learning model, and provide more appropriate recommendations based on the user’s sleep evaluation through top-N recommendations with higher accuracy than traditional filtering techniques. Fusion care is defined by combining user feedback evaluations with sleep data. The preferred sleep pattern is constructed by determining the healthcare service paradigm and data content. Additionally, a personalized profile is created through the service model, which suggests individualized services by analyzing noise and BMI during sleep.

### 3.1. System Architecture of the CDSRS

The proposed system architecture for the CDSRS is shown in Figure 3, below. It consists of three models that incorporate user feedback and a module for processing and managing data that are generated by adjusting the AI motion bed through a mobile UI and creating user profiles based on that experience. The hybrid learning method involves a top-N classification that recommends sleep pattern content as the recommended learning method. The learning method of the inference function processes user sleep pattern evaluations by pattern using the Knn algorithm [34]. In the next step, the recommendation engine provides users with optimal sleep recommendations based on the functions of the learning method according to their evaluations and sleep preferences.

### 3.2. Learning Methods of the Deep Sleep System

The key idea behind the system’s process is to analyze the user profile and preferences by using clustered recommendations in a motion bed environment and to calculate the similarity index between the user and sleep patterns based on feedback evaluations to achieve optimization. The CDSRS is composed of various features and learning methods, as shown in Figure 3.

The algorithm used in the data analysis and user profile server is the K-means clustering technique, which is used for pattern analysis because there is no similarity between sleep data (snoring, noise, BMI, sleep time). The process involves two steps: finding the K-value using the Elbow Method in step 1 and verifying the appropriate K-value using the silhouette validation algorithm in step 2 [35].

Table 1 describes the definition of the algorithm, including specific parameters. 

**Figure 4:**
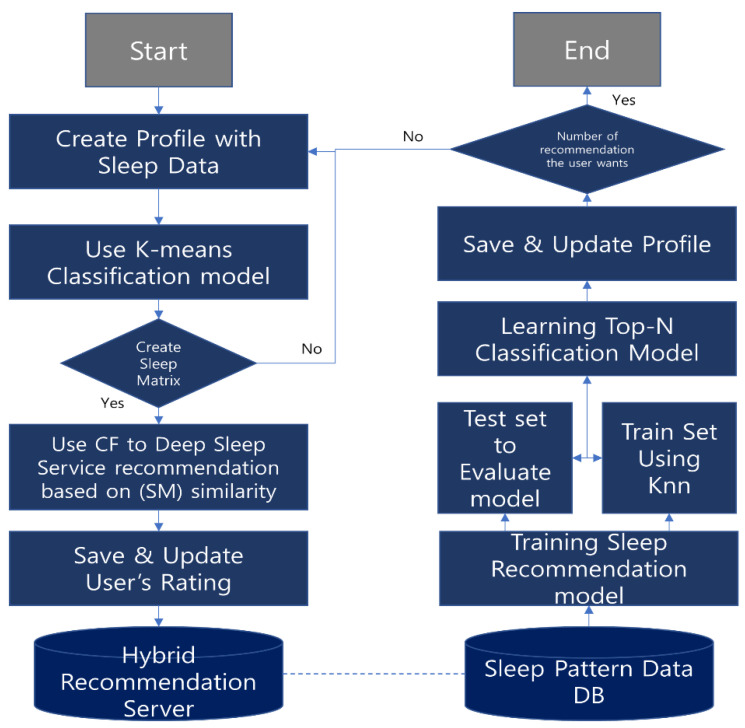
Flow chart of CDSRS algorithm.

The process shown in Figure 4 suggests sleep to the user based on a predefined pattern. First, we create a profile using the user’s sleep data. We then create a sleep matrix with the K-means classification model for the classification of less relevant sleep data such as BMI, sleep time, and snoring. If the profile is created, you will continue to the next step, but if not, you will reconfigure the profile. The CF model is then used to make recommendations, receive user feedback, and reconstruct profiles using hybrid learning methods. After that, a test is conducted with a learning and evaluation model using Knn, and finally, the output is received as a top-N classification model. If the result of the algorithm obtains the list you want, the algorithm ends; otherwise, it goes back to the profile configuration step.

**Figure 5:**
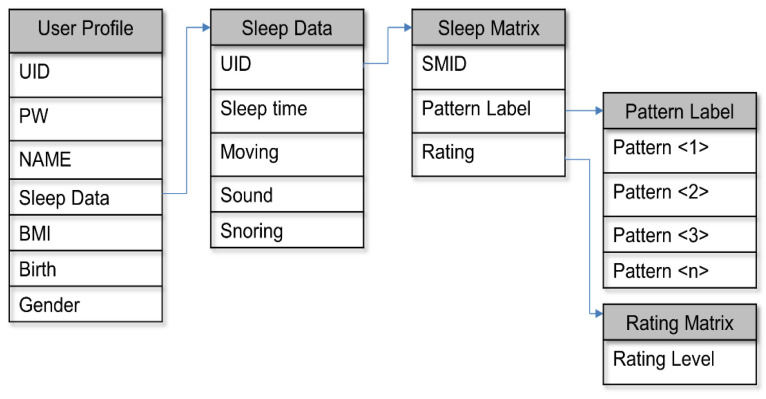
User profile structure.

The CDSRS algorithm consists of the following. The input value is the user’s sleep data, which contains data such as sleep time, moving, sound, and snoring; see Figure 5 for more information. The output value is a list of recommendations from the top-N classification of hybrid learning methods. In this case, the user can control the motion bed by selecting the desired item from the recommendation list. The first of the algorithms constructs a user’s profile with the user’s sleep data. Then, it uses the K-means classification model to create a sleep matrix in a similar group. When a sleep matrix is created, it uses a CF algorithm to calculate similarity and provide recommendation services. Otherwise, it returns to the profile configuration step. In this process, you will receive an evaluation and add it to your profile, gaining sleep pattern analysis data for hybrid learning. Once the appropriate values are obtained, we test them using the evaluation model and, finally, train the top-N classification model. However, the obtained Nan values will be retrained with the Knn algorithm. The last return value is the top-N recommendation you set, and if the number of recommendations you want is achieved, the process ends; otherwise, it returns to the profile configuration step. The process of the CDSRS algorithm is as follows in Algorithm 1.
**Algorithm 1:** CDSRSInputUser’s sleep dataOutputTop-N Categorized Recommendation List1.Create profile with Sleep Data2.Use K-means Classification model3.IF System create Sleep Matrix (SM) with Sleep Data4.  then, Use CF to Deep Sleep Service recommendation based on (SM) similarity5.  Save & Update User’s Rating6.Else: Back to line 27.Training Sleep Recommendation model8.IF System have recommendation results9.  then, Test set to Evaluate model10.  If Loss value is Nan:11.   then, Train Set Using Knn12.   Back to line 1013.  else:14.   Learning Top-N Classification Model15.   return (Top-N Sleep Recommendations)16.Else: 17.  Train set Using Knn18.  Test set to Evaluate model19.  If Loss value is Nan:20.   then, Train Set Using Knn21.  else:22.   Learning Top-N Classification Model23.   return (Top-N Sleep Recommendations)24.Save & Update Profile25.IF Top-N Sleep recommendations == User wants26.  then, Process End27.  return (Top-N Sleep Recommendations)28.Else: Back to line 1

In lines 1 to 2, Algorithm 2 integrates information such as BMI, noise, etc., to calculate similarity and improve patterns. In lines 3 to 5, we repeat the similarity calculation between the sleep matrix (SM) and the user (U) to configure the user-defined profile using Algorithm 3. The resulting data are no longer independent, and this method is called hybrid learning, which combines profile and user evaluation results. This hybrid learning method solves the cold start problem for new users.

Finally, in lines 9 to 24, the user’s assessment of the proposed sleep recommendation is used by Algorithm 4 to analyze the data into a top-N classification. This learning method reconstructs user profiles and excludes less relevant sleep data. This allows you to improve your personalized recommendations through repeated feedback assessments.
**Algorithm 2:** CSPD (Clustering for Sleep Pattern Definition)InputUser’s sleep dataOutputsum_of_squared_distance, k1.sum_of_squared_distance = []2.for k = 1, k in range(1,9) do3.  $\mathrm{sum}\_\mathrm{of}\_\mathrm{squared}\_\mathrm{distance}=\sum_{i=1}^{1}\mathrm{dist}{\left(x,\mathrm{ai}\right)}^{2}$;4. return (sum_of_squared_distance, k)5.$S(i)=\frac{\left(b_{i}\;-\;a_{i}\right)}{\max(a_{i},b_{i})}$6.$a(i)=\frac{\sum_{n}C_{n}i}{n}$$;\;b(i)=\frac{\sum_{n}D_{n}-i}{n}$;7.def i in X,C,D:8. $\mathrm{If}\;a(i)\;<\;b(i),\;s(i)=1-\frac{a(i)}{b(i)}$
9. $\mathrm{If}\;a(i)\;>\;b(i),\;s(i)=\frac{b(i)}{a(i)-\;1}$
10. Label = KMeans(n_clusters = k)11. return (s(i),k)

**Algorithm 3:** PUSA (Prediction User Similarity Algorithm in Sleep Pattern Label)Inputsim(U_1_,U_2_),SM(Sleep Rating Matrix)Outputsim_score1.

$\mathrm{def}\;\mathrm{sim}\_\mathrm{score}(U_{1},U_{2},\mathrm{SM}):\;\mathrm{sim}(U_{1},U_{2})=\frac{\sum_{1}^{n}{(U1}_{i}\;\times \;{U2}_{i})}{\sqrt{\sum_{i}^{n}{\left({U1}_{i}\right)}^{2}\;\times \;\sum_{i}^{n}{\left({U2}_{i}\right)}^{2}}}$

2. If label_n_ not in [SM]:3.   Break;4. else:5.   Return sum([pow(RatingMatrix [U_1_][SM]–RatingMatrix [U_2_][SM],2)])6. For label_n_ in RatingMatrix [U_1_]7.   If label_n_ in RatingMatrix [,U_2_]8.    then, Ignore score sim < 19. Return (sim_score)

**Algorithm 4:** PHRA(Prediction of Hybrid Learning Top-N Rating Algorithm)InputRatingMatrixOutputTop-N recommendation list1.RatingMatrix = Sleep Pattern Rating Matrix2.

$\mathrm{def}\;\mathrm{Top}-N\_\mathrm{prediction}(R,{\mathrm{sim}}_{i}):\;R_{\mathrm{predict}}=\frac{\sum_{i=1}^{N}{\mathrm{sim}}_{i}R_{i}}{\sum_{i=1}^{N}{\mathrm{sim}}_{i}}$

3.   For U in RatingMatrix:4.    if U not in RatingMatrix5.     Break6.    else:7.     result [RM] = R_predict_8.     return result9.Sort list (RatingMatrix SM list)10.Return (Top-N recommendation list)

#### 3.2.1. User Profile Configuration

The profile module consists of pattern analysis, profile management, and feedback reflection. Each module is composed of a user profile and sleep content that reflects the user’s sleep time, BMI, snoring measurement, and noise level during sleep. This basic step helps generate recommendations for personalization and sleep preference collection related to the learning method. Figure 5, below, illustrates the profile structure, explaining the collection and analysis of user data, which helps create customized sleep recommendation content.

First, the user file consists of the user’s ID data, UID, PW, name, sleep data, BMI, birth, and gender. Sleep data are collected through the bed and consist of sleep time, moving, sound, and snoring. For personalized referral systems, we preprocess the UID to configure the sleep matrix and reconstruct it into SMID, pattern label, and rating data. Finally, the algorithm constructs the data in the rating matrix based on the pattern label and the user evaluation.

The creation of a profile is a core feature of personalized sleep recommendation services, which is aided by the learning method. Users can evaluate the first recommended sleep and choose recommendations for sleep content through analysis. This allows users to agree with the analysis of their profile information, enabling the process to be repeated and providing more appropriate content.

With vast amounts of data, classification and definition must be accurately determined, as ambiguous and unrelated data can lead to unclear analysis results. This can result in a loss of meaning and features even if users are classified. Therefore, evaluation and reflection are added to the profile construction process to ensure an accurate optimization process. This reconstruction of the profile helps with user satisfaction and the processing of the profile.

#### 3.2.2. Cold Start Problem

The cold start problem refers to the situation where a recommendation system cannot provide appropriate recommendations to new users because of insufficient pre-existing information. To solve this problem, the CDSRS uses a hybrid learning approach to reconfigure the user’s profile through feedback, even for new users, so that appropriate recommendations can be made.

#### 3.2.3. Defining User Sleep Patterns

The sleep data collected with the AI bed consists of snoring, noise during sleep, BMI, and sleep duration. However, given the nature of sleep data, there is a lack of correlation between the variables. This can lead to inadequate recommendations even after classifying the data. Therefore, we use the K-means algorithm to create meaningful correlations. The K-means algorithm is used to classify data into k clusters, which define the user’s sleep patterns and help find similarities between users and predict additional patterns.

The pseudocode below describes the process of defining sleep patterns, which has a significant impact on recommendations. First, lines 1 to 4 calculate the distance using sleep data and determine the number of centroids (k-value). Lines 5 to 10 go through the silhouette validation process to derive an appropriate k-value out of multiple values. Line 11 returns the silhouette value and the k-value.

**Figure 6:**
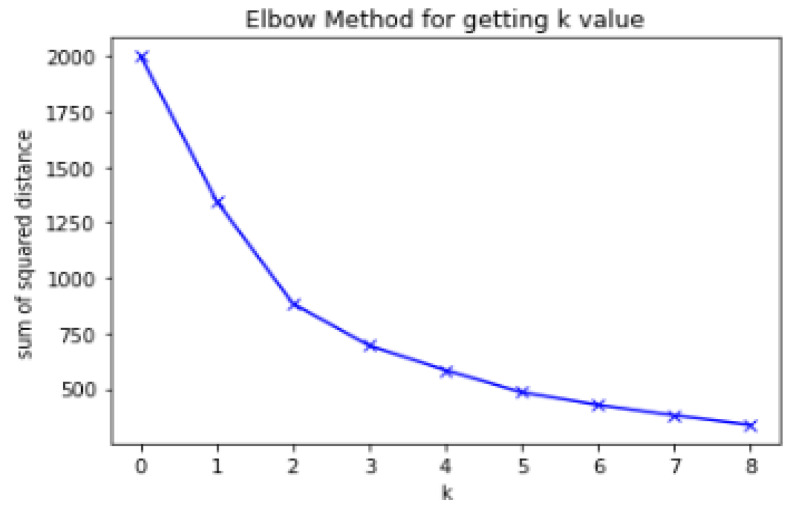
K-values for sleep pattern labels.

Figure 6 shows a graph of changes in the k-value according to the sum of squared distances. We judge that the appropriate k-value is around three to five, and to verify this, the silhouette validation method is used. At this point, the k-value is defined as the number of sleep patterns.

**Figure 7:**
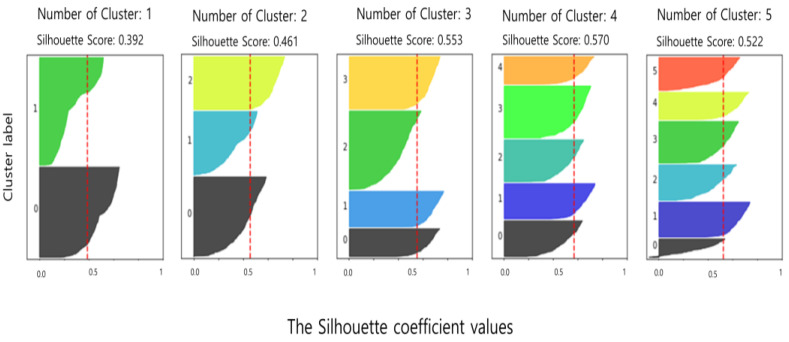
Silhouette verification for appropriate k-values.

Figure 7 represents the process of determining the appropriate number of sleep patterns before recommending similar sleep to users. The silhouette score increases or decreases as the value of k increases. When the k-value is five, the optimal silhouette score is obtained, and it is confirmed that it decreases when the k-value is six, so the efficiency is best when the number of clusters is five.

Through the above process, user profile information is generated. By clustering the data, the meaning of the data becomes clearer, and user satisfaction can be confirmed through recommendations.

#### 3.2.4. Collaborative Filtering

Collaborative filtering predicts users’ interests based on evaluations and feedback gathered from multiple users. With this method, we increase the similarity between users and generated recommendations using sleep-data-based profiles. The resulting process is conceptually simple. When given a user, we normalize their sleep data, calculate the distance from the cluster centroids, and find the cluster corresponding to the user’s sleep pattern. Then, we recommend similar users from that cluster. The algorithm does not need to compute the similarity between all users. The idea is to recommend the most similar sleep patterns between a specific cluster and its corresponding users. This is performed by constructing a user sleep rating matrix, as shown in Table 2.

PUSA uses the Euclidean distance calculation. However, it can be difficult to compare values with just the Euclidean distance formula. Therefore, the algorithm normalizes the data values to create a rating matrix. Additionally, the algorithm explains how to calculate the similarity between two users in the given user SM. The similarity value ranges from −1 to 1, with 1 indicating high similarity and −1 indicating that the users are dissimilar. The defined sim (U1, U2) is used to calculate the similarity between each SM and the users.

Lines 1 to 5 of Algorithm 3 explain how to calculate the similarity between users using Euclidean distance. Lines 6 to 8 transform the values to be in a range of −1 to 1 to construct an SM (Sleep matrix), which represents the ratings given by all users for the same sleep patterns, as shown in Table 3. In this algorithm, the recommendation is based on distance similarity between users and is used as the default setting for user preferences. Values from other patterns are ignored.

#### 3.2.5. User-Based filtering

User-based filtering considers user preferences and ratings to recommend relevant preferences, bringing in the user’s sleep-related attributes and calculating the similarity with evaluation items provided in the user’s sleep data profile. Conceptually, the algorithm operates by determining the sleep patterns that the user is most likely to rate similarly based on past evaluations. In this scenario, the user-based recommendation system continuously relearns using the user profile. The algorithm equation calculates the predicted score by multiplying the user’s evaluation weight by the sim similarity. This is the algorithm that predicts the recommended sleep patterns and scores using the top-N method based on the user’s rated sleep pattern evaluation, as shown in lines 1 to 7 [36]. The final formula for the learning method is Algorithm 4.

Several models can be used to obtain similarity values within a group of sleep patterns and obtain predicted values using sleep pattern evaluations. This is because we want to assign importance levels that vary according to user evaluations. Sleep data analysis considering user evaluations is related to the user’s sleep time, snoring, and primarily recommended sleep evaluations. Through this, the filtering process can create more accurate similar users compared with the initially calculated similar users. The pseudocode in lines 8 to 9 describes the SM list using the predicted values calculated with user evaluations and similarity. This algorithm is effective in the sleep evaluation matrix, so it is ultimately used to normalize weight evaluations.

The proposed algorithm relies on user feedback and engagement, as it is based on user evaluations for profile processing. The learning method’s model is based on user feedback information and user profile feedback. The personalized inference engine analyzes and processes the sleep group, while the recommendation and the adaptation device are integrated with the inference function. The profile is composed of five attributes: sleep duration, snoring severity, noise, BMI, and five sleep patterns. User-based collaborative filtering is utilized in the recommendation algorithm to address the cold start problem.

## 4. Experiment and Evaluation

To verify the performance of the proposed CDSRS, experiments were conducted to calculate the error values that could be derived from incorrect recommendation results [37]. The calculation of error values is similar to supervised learning. Assuming that there is no correct answer in the experimental environment, the difference between the final predicted value and the actual correct answer is defined as the error value. A high error value means that the prediction is inaccurate. To compare the proposed model, the error values of the single CF and CBF models and the combined CF + CBF model were calculated.

The MAPE calculates the relative proportion of the error to the actual value by dividing the difference between the actual and predicted values by the actual value. Then, the absolute value of this proportion is taken, and the average is calculated. Since the degree of error is expressed as a percentage, it is easy to intuitively understand the performance of the model. The MSE is the value obtained by squaring the difference between the predicted value and the actual value and then calculating the average. As the predicted value deviates more from the actual value, the MSE value increases geometrically.

**Figure 8:**
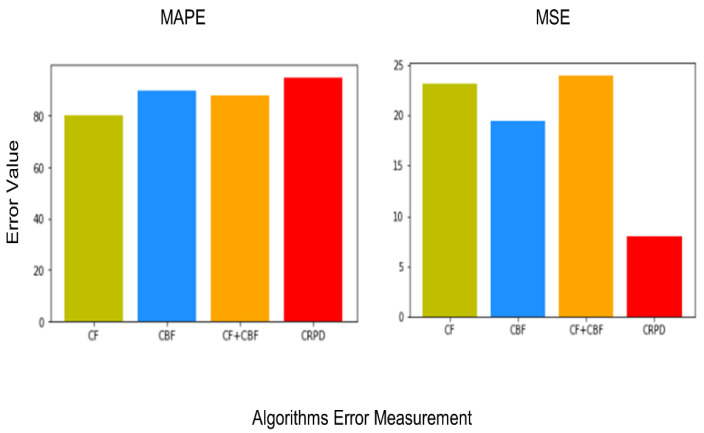
Each experiment’s MAPE/MSE results.

Figure 8 shows the results of the MAPE and the MSE in the Google Keras environment [38]. CF showed the lowest accuracy with 79.5% in the MAPE, and the model combined with CBF had lower accuracy than the CDSRS. The proposed CDSRS had the highest accuracy at 94.2%. CF showed the highest error value with 22.4 in the MSE. CBF improved by about three compared with CF, and the combined model showed an increased error value. The proposed CDSRS had the lowest error value at 9.2, and it was improved by 12.2 compared with CF.

To evaluate the recommendation system, the proposed recommendations to the user include sleep patterns labeled with a sleep label and an estimated sleep rating. To assess the performance of the CDSRS, it must be tested using different machine learning approaches and compared in terms of performance. The CF and CBF algorithms are combined into a single algorithm, CF + CBF, to make predictions. Therefore, the proposed CDSRS method calculates the accuracy and reliability of the performance comparison based on two evaluation criteria. The MAPE has a range of 0–100%, as it is an accurately calculated percentage. The CPRDS had an accuracy of 95.4%, making it the closest to the benchmark of 100%.

Next, we calculate the accuracy of the recommendation using the MSE. The calculation of error using the MSE is similar to calculating the error value using the MAE. However, unlike the MAE, squared operations are performed to weight higher error values. This means that higher error values are given more weight, while lower error values are given less weight.

To evaluate the performance of the CDSRS learning algorithm, two evaluation criteria, the MSE and the MAPE, were used. As shown in Table 4 and Table 5, the CDSRS exhibited a relatively high value of MAPE at around 10% compared with other models, while the accuracy value of the MSE model decreased by approximately eight, indicating that it is the most stable and improved average model. The proposed CDSRS can analyze and predict sleep data with low correlation as patterns and recommend sleep patterns using ML by incorporating user changes. As shown in Table 6 and Table 7, the experimental results demonstrate that the proposed CDSRS outperforms the combination CF, CBF, and CF + CBF models in terms of performance.

## 5. Conclusions and Future Works

This paper introduces a hybrid deep learning approach to construct a personalized sleep service recommendation system. The proposed system utilizes K-means clustering to analyze sleep data with low correlation and identify similar sleep patterns, which are then used to create user profiles. User feedback from the top-N-rated process is employed to reconstruct the profiles. The system leverages this feedback and user sleep data for learning purposes and subsequently generates and releases the final recommendations in the form of a top-N list. This hybrid learning approach effectively addresses the cold start problem and enhances the efficiency and accuracy of the recommendations. Furthermore, a matrix combining sleep patterns and user ratings is employed as a variable to improve the model’s accuracy.

To evaluate the performance of the proposed model, 1000 user data points were considered, and K-means clustering was employed to define five sleep patterns. The sleep data used in this study consist of the user’s UID, BMI, snoring, sleep time, noise, and movement. The mean squared error (MSE) and mean absolute percentage error (MAPE) were used for error verification. Based on the MSE values, the CDSRS model exhibited 13.2% less error compared with the collaborative filtering (CF) model and 10.2% less error compared with the content-based filtering (CBF) model. Regarding the MAPE, the CF model achieved 79.5% accuracy, while the CBF and CF + CBF models achieved 85% accuracy. Notably, the proposed CDSRS model demonstrated the highest accuracy, reaching 94.2%. Traditional methods do not reflect user sleep assessments, and there is no way to improve the accuracy of recommendations as the number of users increases, but CDSRS can solve existing problems using advanced results and hybrid methods, allowing users to make more accurate recommendations and provide customized services.

In future studies, CDSRS can combine EEG (EEG) data. EEG refers to an electrophysiological measurement method that records the electrical activity of the brain through electrodes. This has the potential to increase our understanding of sleep stages and provide more accurate and personalized services. EEG patterns allow research to identify specific sleep stages that an individual is experiencing, allowing RSs to deliver more accurate, customized recommendations. In addition, real-time sleep monitoring can be integrated to improve the accuracy of personalized recommendations by providing the system with the user’s sleep state. It can also be replaced by feedback, such as surveys, instead of user feedback methods using traditional ranking systems. These systems can leverage natural language processing models to provide more diverse and sophisticated recommendations.

## Figures and Tables

**Table 1:** Sleep research trends.

Year	Sleep Research
2001	A microwave radio for Doppler radar sensing of vital signs;
2008	Video-based activity and movement pattern analysis in overnight sleep studies;
2011	Comparison of center estimation algorithms for heart and respiration monitoring with microwave Doppler radar;
	Pulse pressure monitoring through non-contact cardiac motion detection using 2.45 GHz microwave Doppler radar;
2013	Noncontact screening system with two microwave radars for the diagnosis of sleep apnea–hypopnea syndrome;
2014	Monitoring and analysis of respiratory patterns using microwave Doppler radar
2016	Personal recommendation system for improving sleep quality;
2020	Personalized user modeling for context-aware lifestyle recommendations to improve sleep;
2022	Sleep posture recognition with a dual-frequency microwave Doppler radar and machine learning classifiers.

**Table 2:** Parameters.

Parameters	Description
K	Number of sleep pattern labels
Sim (U_a_,U_b_)	Euclidean distance to calculate the similarity between two users
SM	SM_n_, number of sleep labels in rating matrix
Rating Matrix	User’s sleep pattern rating matrix, from −1 to 0 to 1 in the sleep rating matrix
Sleep Rating	One to five ratings of sleep patterns evaluated by users
U	U_n_, number of users in rating matrix
Top-N	N with high accuracy in the algorithm result list
Nan	Error value

**Table 3:** Sleep rating matrix.

	U_1_	U_2_	U_3_	U_4_	**U_n_**
SM_1_	1		1	−1	
SM_2_	−1		−1		
SM_3_	1	1		1	
SM_n_		1		−1	

**Table 4:** Formulas of MSE and MAPE evaluation criteria.

Evaluation Criterion	Formula	Functional
MSE	$\frac{1}{n}{\sum_{1}^{n}(d_{i}-{\hat{d}}_{i})}^{2}$	Mean squared error is a combination measurement of bias and variance of prediction.
MAPE	$\frac{100}{n}\sum_{1}^{n}\frac{d_{i}-{\hat{d}}_{i}}{d_{i}}$	Mean absolute percentage error is similar to MAE but is normalized by a true observation. The downside is that, when the true observation is zero, this metric will be problematic.

**Table 5:** Summary of mean squared error (MSE) values.

Model	The Average Error Value of the Model According to MSE			Proposed Method
MSE	CF	CBF	CF + CBF	CDSRS
	22.4	19.4	23.8	9.2

**Table 6:** Accuracy of recommendations using MAPE.

Model	Mean Error Values of the Models According to MAPE			Proposed Method
MAPE	CF	CBF	CF + CBF	CDSRS
	79.5	86	84.3	94.2

**Table 7:** Comparison of final results.

	Comparison of Final Results			A Proposed Method
Model	CF	CBF	CF + CBF	CDSRS
MSE	22.4	19.4	23.8	9.2
MAPE	79.5	86	84.3	94.2

## Data Availability

The data presented in this study are available on request from the corresponding author.
